# Belief in conspiracy theories and esoteric thinking during COVID-19 pandemic

**DOI:** 10.1192/j.eurpsy.2022.231

**Published:** 2022-09-01

**Authors:** T. Medvedeva, S. Enikolopov, O. Boyko, O. Vorontsova

**Affiliations:** Federal State Budgetary Scientific institution “Mental health research center”, Clinical Psychology, Moscow, Russian Federation

**Keywords:** Depression, Covid-19, esoteric thinking, conspiracy theories

## Abstract

**Introduction:**

Psychological distress during the SARS-CoV-2 pandemic can manifest itself in interpretations of what is happening.

**Objectives:**

To analyze response to COVID-19 pandemic in people with high level of esoteric thinking.

**Methods:**

Internet survey 23.03.20-29.01.21 (N=621); Constructive Thinking Inventory(CTI); SCL-90R. It was proposed to assess statements: “The authorities are hiding the true scale of the coronavirus pandemic”, “Coronavirus is the result of biological weapons development”, “Coronavirus is a punishment or a sign sent to people from above”, “The emergence of the coronavirus is the Earth’s response to its pollution”. It was offered to express an opinion about pandemic. The answers were coded on the basis of qualitative semantic analysis.

**Results:**

The growth of “esoteric thinking” was revealed (Std.J-T, p=.025). With a high level of esoteric thinking, emotional statements (“fear, anxiety, panic”) are more common (27.8% versus 16.9% for group with high and low level of «esoteric thinking»). Correlations of the level of «esoteric thinking» with level of depression (Spearman’s correlation ,085*), anxiety (,097*), GSI (,130**), «fears for the life» (23.4% versus 14.5%) show high emotional distress. With an increase in the level of «esoteric thinking», belief in various conspiracy theories increases; Spearman’s correlation ,370** with the belief is biological weapons, punishment for sins (,355**), belief in concealing information about the pandemic (,167**).

**Conclusions:**

A high level of esoteric thinking is associated with an increased emotional response to the pandemic and with belief in conspiracy theories, and can increase emotional instability by itself also making constructive decisions difficult in situations related to protecting personal health and safety.

**Disclosure:**

No significant relationships.

